# Soil phosphorus availability and fractionation in response to different phosphorus sources in alkaline and acid soils: a short-term incubation study

**DOI:** 10.1038/s41598-023-31908-x

**Published:** 2023-04-07

**Authors:** Yuan Wang, Wei Zhang, Torsten Müller, Prakash Lakshmanan, Yu Liu, Tao Liang, Lin Wang, Huaiyu Yang, Xinping Chen

**Affiliations:** 1 College of Resources and Environment, Academy of Agricultural Sciences, Key Laboratory of Efficient Utilization of Soil and Fertilizer Resources, Southwest University, Chongqing, 400716 China; 2 Interdisciplinary Research Center for Agriculture Green Development in Yangtze River Basin, Southwest University, Chongqing, China; 3 Institution of Crop Science, University of Hohenheim, 70593 Stuttgart, Germany; 4 Sugarcane Research Institute, Guangxi Academy of Agricultural Sciences, Nanning, 530007 China; 5 Queensland Alliance for Agriculture and Food Innovation, University of Queensland, 4067, St Lucia, QLD Australia; 6 College of Life Sciences, Zhejiang University, Zhejiang, 310058 China; 7 Chongqing Academy of Agriculture Sciences, Chongqing, 40000 China

**Keywords:** Agroecology, Environmental impact

## Abstract

Using agricultural wastes as an alternative phosphorus (P) source has great prospects to improve soil P status. A 70-day incubation experiment was carried out to investigate the effects of superphosphate (SSP), poultry manure (PM), cattle manure (CM), maize straw (MS), and cattle bone meal (CB) with the same total P input on soil P availability and fractions in typical acidic (red soil) and alkaline (fluvo-aquic soil) soils. The results showed that in both fluvo-aquic and red soils, CM out-performed other P sources in improving soil P availability. Changes in soil Olsen-P (ΔOlsen-P) were greater in fluvo-aquic soils with SSP, PM and CM additions than in red soils. Among the different P sources used, only CM has increased the labile soil P fractions to levels similar to that with SSP. Compared with SSP, more monoester P and inositol hexakisphosphate were detected in soils amended with PM and CM. A structural equation model (SEM) analysis suggested that soil pH had a direct positive effect on the labile P fractions in the acidic red soil amended with different P sources. In summary, CM is a superior P source for increasing plant available soil P, with considerable practical implications for P recycling.

## Introduction

Phosphorus (P) is a key nutrient in intensive agricultural production as it is essential for crop growth and yield^1,2^. However, resource limitation and chemical P-induced environmental pollution are the global challenges present day agriculture^3,4^. Re-adjusting P input, reducing P loss, and recycling P in agricultural wastes such as manure, straw, animal bone meal, etc. are considered to be effective strategies to reduce chemical P inputs globally^5–9^. Understanding the distribution of different inorganic and organic P fractions is a prerequisite for the control of phosphate transformation in soils. Thus, understanding the transformation and availability of soil P fractions following recycling different P sources is particularly important for improving crop P utilization and P fertilizer management while reducing environmental risks.

The physicochemical transformations of P (dissolution, precipitation, adsorption and desorption) are regulated by soil pH, organic matter content and soil biological properties^10–12^. The addition of chemical P fertilizers (superphosphate, SSP) leads to an initial spike in P availability, followed by P adsorption and precipitation, which will result in a substantial decrease in P availability over time^13^. Compared with chemical P, organic fertilizer inputs are beneficial to the conversion of moderately labile P to available P^14^. Alternative P sources have a variety of P compounds, including a large proportion of orthophosphate^15^. These alternative P sources can also affect P kinetics in soil by changing the adsorption capacity^16,17^. The P fractions in manure are dependent on various factors, including manure type, solid–liquid separation status, decomposition rate, and handling processes and storage of manure^18,19^. Also, the differences in the digestive system and feed composition of animals can cause large differences in P concentration and fractions in different manures^20,21^. Previous research suggested that most P in poultry manure was recovered in water and HCl extracts, whereas most P in cattle manure was recovered in NaHCO_3_ extract^22^. Hence, the P availability in cattle manure tends to be higher than that of poultry manure. The transformation of P from various manure types applied to soil warrants further investigations. Moreover, crop straw is usually returned directly to the soil in agricultural practice, and the P availability from straw requires in-depth analysis^23^. The bone meal is proposed to be recycled and used as organic fertilizer, whereas its potential use as an effecient source remains unclear^6^. Thus, it is necessary to identify and quantify P fractions from different alternative P sources and their distribution in soil P fractions to determine the potential P availability.

The relative contents of inorganic P (Pi) and organic P (Po) in soil were greatly affected by soil type, land use and organic amendment sources^24–26^. Soil physicochemical properties such as pH, texture, and organic matter content determined the P sorption reaction^27–29^. It is crucial to reveal the transformation mechanism of different alternative P sources in soil and their relationship with soil properties by studying the difference of P fractions in a typical red soil (low pH) and a fluvo-aquic soil (slightly alkaline pH) with different alternative P sources and their transformation dynamics in soil. Quantifying the transformations of different alternative P sources under different soil conditions is necessary to enhance P utilization and reduce chemical P input.

The improved sequential P fractionation technique allows to separate total soil P into labile P, moderately labile P, sparingly labile P and non-labile P fractions^30,31^. Although soil P sequential fractionation defines soil P fractions based on their solubility, it provides limited information on the biogeochemical processes and plant availability of P. The ^31^P solution nuclear magnetic resonance (NMR) has been widely used to study soil P transformation and can provide better understanding of organic P compounds. Previous studies suggested that both Pi and Po inputs increased orthophosphate and the diversity of P forms^32–34^. In contrast, Annaheim et al. (2015) reported that the long-term addition of organic fertilizers had little effect on soil organic P^35^. The combination of the classical P sequential fractionation with the advanced P speciation analysis technique allows a more powerful approach to studying P turnover processes in soil.

Recycling P from alternative P sources is an important and necessary step for green and sustainable agriculture and a clean environment^36–38^. Fluvo-aquic and red soils are extensively used for agricultural production in China. Compared with fluvo-aquic soil, red soil readily adsorbs P and reduces its availability, necessitating high P fertilizer use in agriculture. Quantifying the temporal variation of soil P availability in different soil types (representative of alkaline and acidic soils) with different P source input requires further investigation. Hence, the aims of this study were (1) to evaluate the impact of different alternative P sources on soil P fractions in fluvo-aquic and red soils, and (2) to reveal the relationship between soil P fractions and P availability. We hypothesized that: (1) cattle manure (CM) is a more efficient alternative P source than poultry manure (PM), maize straw (MS) and cattle bone meal (CB), and that (2) compared with fluvo-aquic soil, red soil tend to increase immobiliation of P from alternative P sources, and (3) the potential availability of P from various sources is determined by the ratio of soil labile P fractions to total P.

## Results

### Soil Olsen-P concentration of alternative P sources

**Figure 1:**
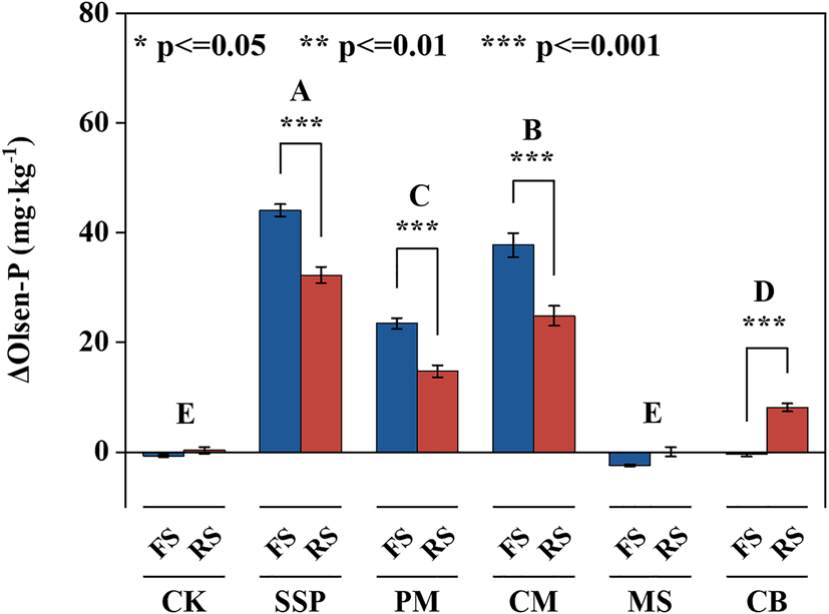
Changes in Olsen-P concentrations (ΔOlsen-P) in fluvo-aquic and red soils supplemented with different phosphorus sources during 70-day incubation. Values are means ± SE (n = 4). FS: Fluvo-aquic soils; RS: Red soils; CK: Control; SSP: Ca(H_2_PO_4_)_2_; PM: Poultry Manure; CM: Cattle Manure; MS: Maize Straw; CB: Cattle Bone Meal. Dissimilar uppercase letters denote significant differences among the P sources (*P* < 0.05); * indicate significant differences between the fluvo-aquic soil and red soil. *, ** and *** indicate significant at *P* < 0.05, *P* < 0.01 and *P* < 0.001, respectively. The error bar represents SE.

The changes in soil Olsen-P (ΔOlsen-P) was significantly affected by P source and soil type (Fig. 1). In both soils, the Olsen-P fluctuated with incubation time (Fig. S2). Soil P availability in response to P sources decreased in the order of SSP > CM > PM > CB > MS ≥ CK. The Olsen-P of two soils amended with SSP, PM, CM and CB increased by 38.4, 19.3, 31.5 and 4.03 mg kg^−1^ respectively, compared with CK. CM outperformed other P sources in increasing Olsen-P concentration. In both soils, CM significantly increased Olsen-P by 12.2, 32.5, and 27.4 mg kg^−1^ compared with PM, MS and CB, respectively. The ΔOlsen-P of the red soil in response to SSP, PM and CM additions were decreased by 11.9, 8.7 and 12.9 mg kg^−1^ compared to that in fluvo-aquic soil, respectively. However, the Olsen-P of the red soil in response to CB additions was increased by 8.6 mg kg^−1^ compared to that in fluvo-aquic soil.

### Soil P sequential fractionations in response to P sources

**Figure 2:**
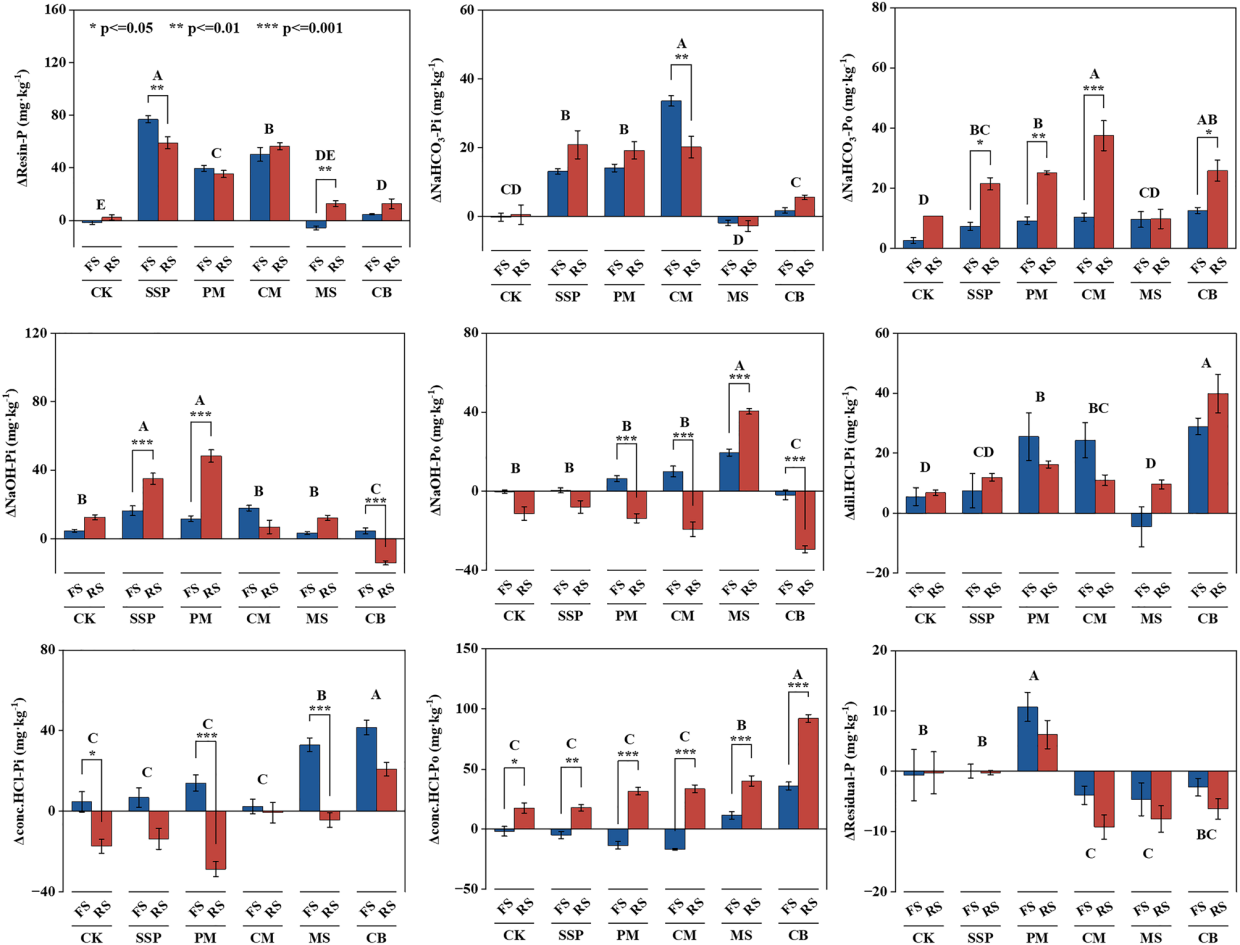
Changes in P fractions (ΔP fractions) in fluvo-aquic and red soils amended with different phosphorus sources on DAI 70. Values are means ± SE (n = 4). FS: Fluvo-aquic soils; RS: Red soils; CK: Control; SSP: Ca(H_2_PO_4_)_2_; PM: Poultry Manure; CM: Cattle Manure; MS: Maize Straw; CB: Cattle Bone Meal. Dissimilar uppercase letters denote significant differences among the P sources (*P* < 0.05). * indicate significant differences between the fluvo-aquic soil and red soil. *, ** and *** indicate significant at *P* < 0.05, *P* < 0.01 and *P* < 0.001, respectively. The error bar represents SE.

Amendment of alternative P sources to the fluvo-aquic and red soils significantly altered soil P fractions on DAI 70 (Table S3). The changes in soil P fractions (ΔP fractions) was significantly affected by P source and soil type (Fig. 2). The soil labile P fractions (Resin-P + NaHCO_3_-Pi + NaHCO_3_-Po) of two soils amended with SSP, PM, CM and CB increased by 92.5, 64.2, 97.0 and 24.5 mg kg^−1^ respectively, compared with CK. Among different alternative P sources, CM was the only source that had increased labile soil P fractions to levels similar to that in SSP. Additions of alternative P sources had also increased the moderately labile P concentrations in the soil. The soil moderately labile P fractions (NaOH-Pi + NaOH-Po + dil.HCl-P) of two soils amended with SSP, PM, CM and MS increased by 22.4, 38.2, 16.2 and 31.2 mg kg^−1^ respectively, compared with CK. MS and CB additions significantly increased the soil sparingly labile P fractions (conc.HCl-Pi + conc.HCl-Po) of two soils by 38.5 and 93.6 mg kg^−1^ compared with CK.

The ΔResin-P and Δconc.HCl-Pi of the red soil in response to SSP additions were decreased by 118.3 and 20.3 mg kg^−1^, but increased by 14.0, 18.7 and 23.0 mg kg^−1^ for ΔNaHCO_3_-Po, ΔNaOH-Pi and Δconc.HCl-Po, compared to that in fluvo-aquic soil. The ΔNaHCO_3_-Po, ΔNaOH-Pi and Δconc.HCl-Po of the red soil in response to PM additions were increased by 16.0, 36.3 and 45.0 mg kg^−1^, but decreased by 20.0 and 42.7 mg kg^−1^ for ΔNaOH-Po and Δconc.HCl-Pi, compared to that in fluvo-aquic soil. The ΔNaHCO_3_-Pi and ΔNaOH-Po of the red soil in response to CM additions were decreased by 13.7 and 29.3 mg kg^−1^, but increased by 27.3 and 50.7 mg kg^−1^ for ΔNaHCO_3_-Po and Δconc.HCl-Po, compared to that in fluvo-aquic soil. The ΔResin-P, ΔNaOH-Po and Δconc.HCl-Po of the red soil in response to MS additions were increased by 18.0, 20.7 and 28.7 mg kg^−1^, but decreased by 37.0 mg kg^−1^ for Δconc.HCl-Pi, compared to that in fluvo-aquic soil. The ΔNaHCO_3_-Po and Δconc.HCl-Po of the red soil in response to CB additions were increased by 13.0 and 56.3 mg kg^−1^, but decreased by 18.7 and 27.7 mg kg^−1^ for ΔNaOH-Pi and ΔNaOH-Po, compared to that in fluvo-aquic soil.

### Changes in soil Po compounds in response to P sources

**Figure 3:**
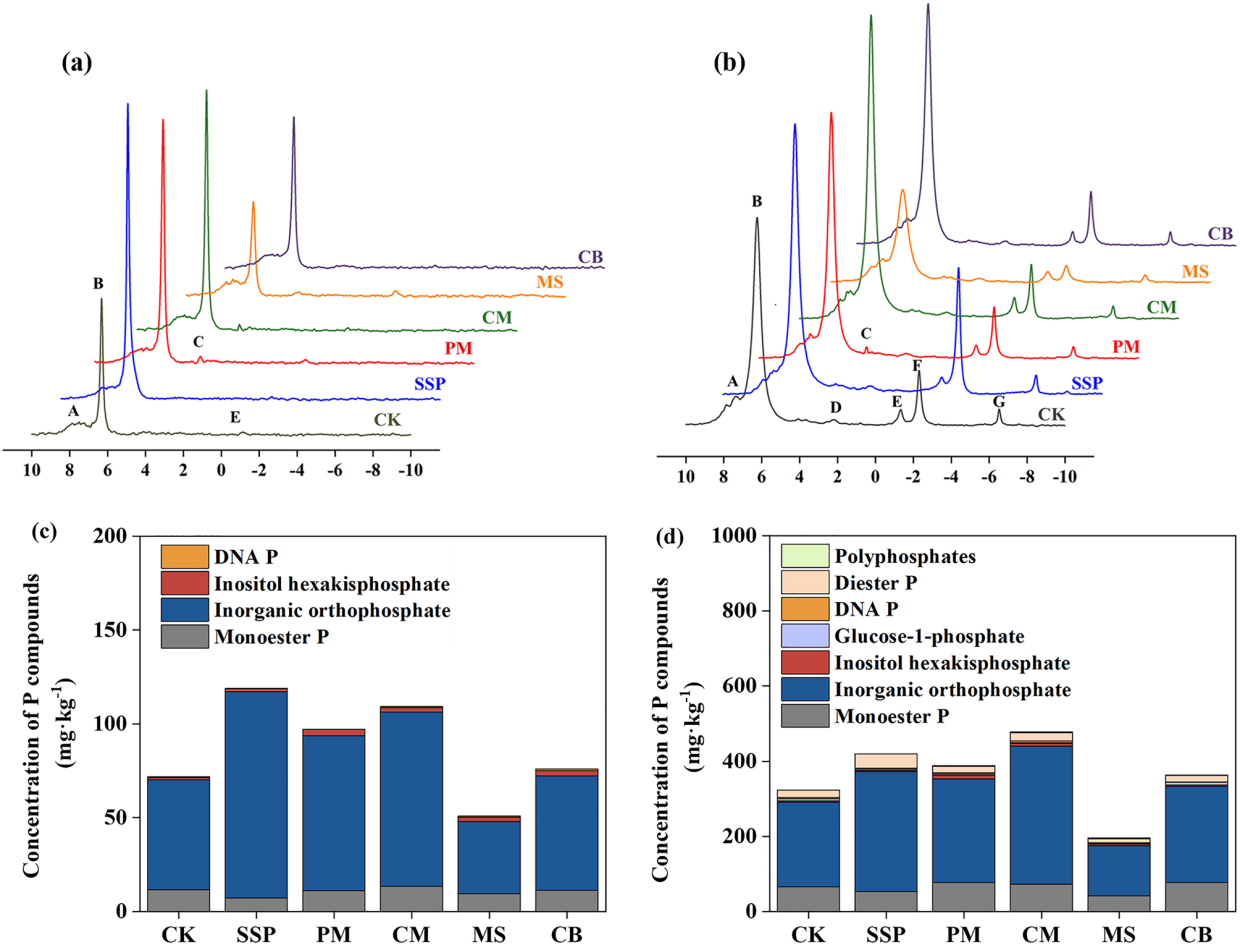
Liquid ^31^P NMR spectra of NaOH-EDTA extracts of fluvo-aquic (**a**) and red soils (**b**) amended with different alternative P sources. Concentrations of P compounds in NaOH-EDTA extracts of fluvo-aquic (**c**) and red soils (**d**) by ^31^P-NMR. In the upper spectrum, the shift positions of the different P compounds are indicated. A: Monoester P (7.19 to 7.58 ppm); B: Inorganic orthophosphate (6.18 to 6.34 ppm); C: Inositol hexakisphosphate (4.38 to 4.49 ppm); D: Glucose-1-phosphate (3.13 to 3.43 ppm); E: DNA P (− 0.15 to − 0.36 ppm); F: Diester P (− 1.73, − 2.43 ppm); G: Polyphosphates (− 4.63 to − 5.83 ppm). CK: Control; SSP: Ca(H_2_PO_4_)_2_; PM: Poultry Manure; CM: Cattle Manure; MS: Maize Straw; CB: Cattle Bone Meal.

The ^31^P-NMR spectra recorded clear peaks in the Po and Pi regions, including monoester P, inorganic orthophosphate, inositol hexakisphosphate, glucose-1-phosphate, DNA P, diester P and polyphosphates (Fig. 3a,b). Irrespective of the soil type, inorganic orthophosphate was the dominant P compound in NaOH-EDTA extracts. In fluvo-aquic soil, inorganic orthophosphate concentrations ranged from 38.5 to 110.0 mg kg^−1^ for all the treatments and the peak value of SSP was the highest, followed by that of CM. In red soil, inorganic orthophosphate concentrations ranged from 165.3 to 1674.6 mg kg^−1^ for all the treatments and the maximal value was recorded in CM treatment, followed by SSP, which were consistent with the changes in Olsen-P and labile P concentrations (Figs. 1 and 2). Compared with SSP treatment, monoester P and inositol hexakisphosphate contents were higher in soils amended with PM and CM (Fig. 3c,d).

### Effect of soil pH and TOC on P fraction transformation in response to different P sources

**Figure 4:**
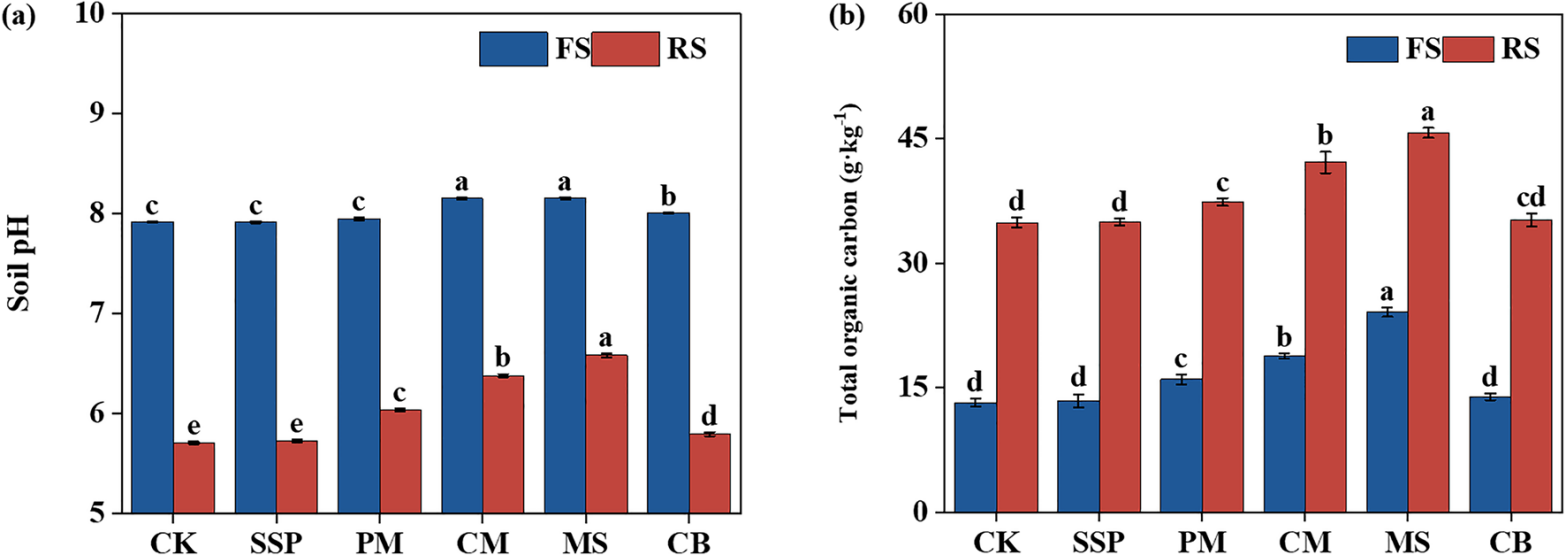
Soil pH (**a**) and total organic carbon (TOC) (**b**) of fluvo-aquic and red soils with different alternative P sources on DAI 70. FS: Fluvo-aquic soils; RS: Red soils; CK: Control; SSP: Ca(H_2_PO_4_)_2_; PM: Poultry Manure; CM: Cattle Manure; MS: Maize Straw; CB: Cattle Bone Meal. Different lowercase letters indicate significant differences among the P sources. Significant at *P* < 0.05. The error bar represents SE.

CM and MS additions significantly increased the pH of both soils (Fig. 4a). In fluvo-aquic soil, CM and MS additions increased the pH by 3.0%. In red soil, PM, CM and MS additions increased the pH by 4.2%, 8.6% and 11.2%, respectively. PM, CM and MS additions also significantly increased TOC content of both soils (Fig. 4b). Compared with CK, application of PM, CM and MS increased the fluvo-aquic soil TOC by 21.2%, 42.7% and 82.6%, respectively. Similarly, TOC of PM-, CM- and MS-supplemented red soils were 7.1%, 20.8% and 30.9%, respectively, greater than that of CK.

**Figure 5:**
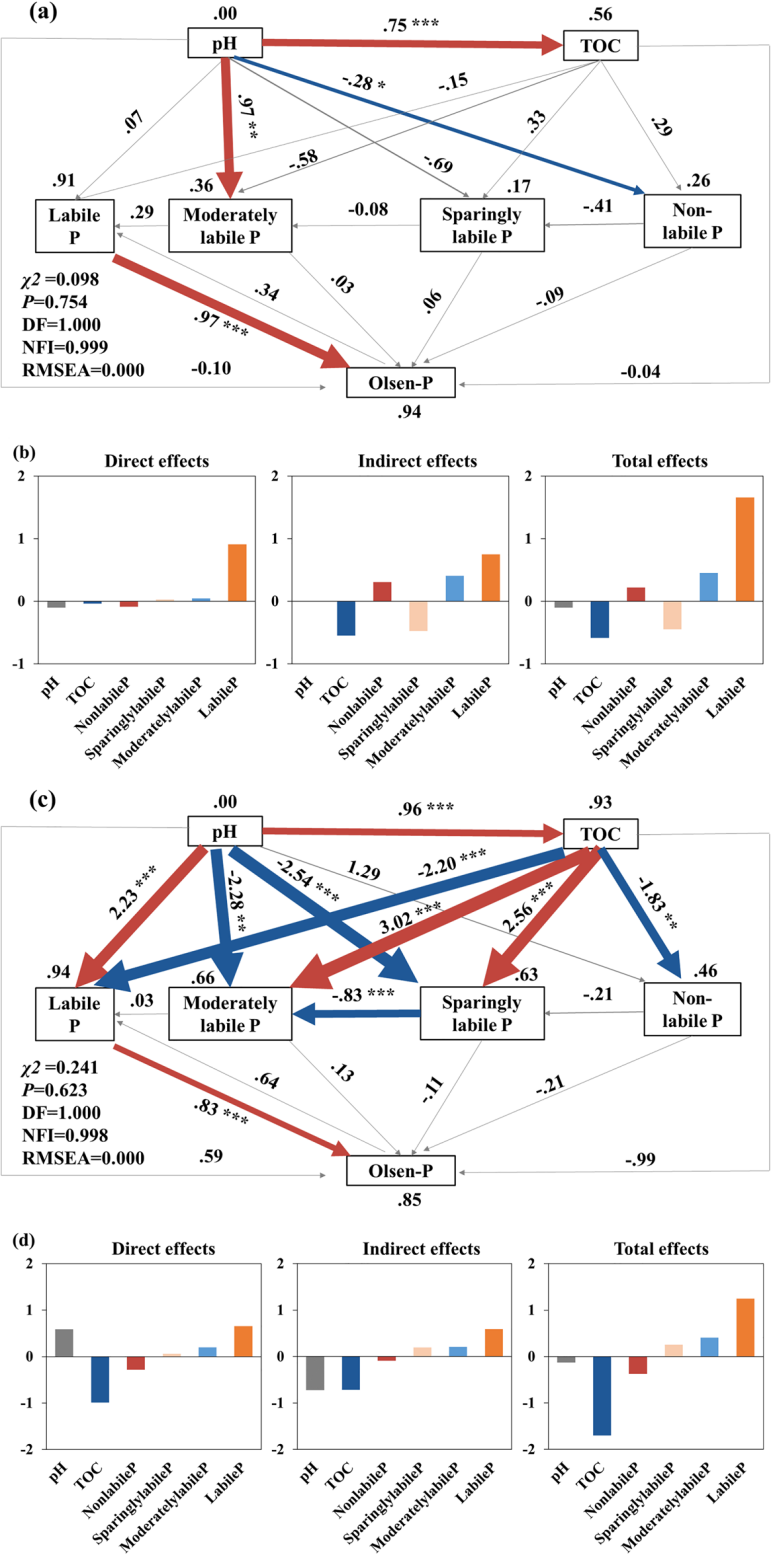
Structural equation model (SEM) analysis for the transformation of P fractions in response to the addition of different alternative P sources in fluvo-aquic (**a**) and red soils (**c**). Standardized direct effects, indirect effects and total effects of the factors on Olsen-P from the structural equation modeling model of fluvo-aquic (**b**) and red soils (**d**). Optimal model fitting results under the fluvo-aquic soil (**a**): χ^2^ = 0.098, DF = 1, χ^2^/DF = 0.098, *P* = 0.754, NFI = 0.999 and RMSEA = 0.000; optimal model fitting results under the red soil (**b**): χ^2^ = 0.241, DF = 1, χ^2^/DF = 0.241, *P* = 0.623, NFI = 0.998 and RMSEA = 0.000. The number on the arrow represents the standardized path coefficient, and the red and blue arrows represent the positive and negative effects, respectively. *, ** and *** indicate significant at *P* < 0.05, *P* < 0.01 and *P* < 0.001, respectively. The black number above each variable is R^2^ values, which represent the explained proportion of variance for each variable. The arrow width indicates the strength of the paths.

Effects of soil properties on soil P fractions and Olsen-P concentration were further analyzed using the SEM (Fig. 5). Both soil pH and TOC had no significant effect on labile P fractions of fluvo-aquic soil. However, soil pH showed a significantly positive effect on moderately labile P fractions of fluvo-aquic soil (path coefficient was 0.97). In contrast, for red soil, pH showed a significant positive effect on labile P fractions (path coefficient was 2.23) but negative effects on moderately and sparingly labile P fractions (path coefficients were − 2.28 and − 2.54, respectively). Increasing TOC significantly reduced labile and non-labile P fractions of red soil (path coefficients were − 2.20 and − 1.83, respectively), whereas increased the moderately and sparingly labile P fractions (path coefficients were 3.02 and 2.56, respectively). Overall, the variables explained 94.0% and 85.0% of Olsen-P variation in fluvo-aquic soil and red soil, respectively. In both soils, the labile P fractions had a direct positive effect on soil Olsen-P (path coefficients were 0.97 and 0.83, respectively), implying that the soil P availability was driven by the labile P fractions.

## Discussion

### Variability of soil P availability in fluvo-aquic and red soils amended with alternative P sources

The SSP treatment resulted in the highest Olsen-P concentration in both fluvo-aquic and red soils (Fig. 1 and Fig. S2). Chemical P (SSP) can be readily dissolved in soil solution and immediately transformed with labile soil P fractions. In contrast, Po in manure needs to be mineralized to dissolve in soil solution^36,39^. Therefore, the P availability of both soils was higher with SSP than with alternative P sources. This study demonstrates that CM has an inherent advantage in increasing soil P availability compared to other alternative P sources. Manure type is a key factor which affects P components, and manure type has a profound effect on soil P fractions^40^. The differences in the digestive system of animals and in the composition of the feed may result in significant differences in the P content and its fractionation among manures, which in turn affects the accumulation, transformation and mobility of P in the soil and affects its bioavailability^41,42^. Previous study has shown that the extractability of Olsen P from CM amendments is higher than that from PM^43^. This is consistent with the results of the current study. Moreover, other studies suggest that long-term straw residual return to the soil improved soil available P^44,45^. However, in the current study, incorporation of MS in the soil did not improve soil P availability in both soils. In long-term field studies, the addition of organic manure rather than crop straw influenced soil P availability^46,47^. The type of straw and the processing method may affect its soil enrichment capacity. Thus, future research should consider the management method of straw returning to the field to promote its in situ decomposition and nutrient release to improve soil available P.

The extent of soil P fraction variation following the application of organic amendments depends to a large degree on soil type and texture^48^. Compared with the fluvo-aquic soil, the red soil showed higher P adsorption capacity following SSP, PM and CM amendments (ΔOlsen-P decreased). Similarly, Yang et al. (2013) suggested that the increase of Olsen-P concentrations was higher in the slightly alkaline soil than that in acid red soil following organic amendments^49^. Further, CB addition improved soil P availability in the red soil, but not in the fluvo-aquic soil. This could be attributed to that P mainly exists in the form of apatite in bone meal, and H^+^ is necessary to release P from apatite^6,50^. This suggests that the application of CB as a source of P fertilizer in acidic red soil is more promising than that in fluvo-aquic soil.

### Characterization of soil P fractions of fluvo-aquic and red soils with alternative P sources

Soil P fractions analysis revealed the rapid transformation of most of the chemical P into the labile P fractions (Table S3). However, alternative P sources mainly increased the moderately and sparingly labile P concentrations in both soils. Correlation analysis showed a significantly positive correlation between soil P availability and labile P fractions (Fig. S4). The proportion of P to soil labile P fractions differed among alternative P sources, which could be the main reason for the variation in available P concentration in both soils. Comparing the P fractions of the alternative P sources, the labile P fractions of poultry manure, cattle manure, maize straw and cattle bone meal accounted for 47.5%, 66.7%, 7.3% and 9.1% of the total P, respectively (Table S2). Therefore, CM outperformed other sources in improving soil P availability. Numerous studies have proved that the type of animal manure has a strong effect on the P fractions. Previous studies have shown that most of the P in PM is extracted by water and HCl, while most of the P in CM is extracted by NaHCO_3_^18^. With equal amounts of manure P inputs, CM with a higher percentage of labile P may have higher soil P availability than PM. This is consistent with the findings that Olsen-P concentrations were higher in CM amended treatments for both soils. In contrast, mineralization of other P sources such as MS and CB were relatively slow to release available P. These results imply that MS and CB are poorly involved in the soil P cycle over a short time. Therefore, fluvo-aquic soil with MS and CB did not show a significantly beneficial effect on soil P availability in the short term.

The addition of SSP and CM to the red soil significantly decreased the ΔResin-P and ΔNaHCO_3_-Pi, respectively, compared with that to fluvo-aquic soil. P adsorption is mainly dominated by iron (Fe) and aluminum (Al) hydroxides and clay minerals in acid soils^51^. In contrast, the effects of CaCO_3_ and Ca-phosphates precipitation were more strongly in neutral and slightly alkaline soils^26^. The Fe and Al ions and hydroxides in red soil increase the sorption and decrease the decomposition of Po, thereby inhibiting the conversion of moderately labile P to labile P fractions^52^. Consequently, the accumulation of P in labile P fractions in red soil is less than that in fluvo-aquic soil, which could have reduced P availability in soil. The change in soil pH caused by fertilization will affect the adsorption and desorption of P in soil. In acidic soils, the increase of pH and the decrease of extractable Al compounds can reduce the P adsorption of the soil^53,54^. On the contrary, in fluvo-aquic soil, the Ca-phosphates precipitation may increase with increasing pH^55^. The SEM indicated that increasing soil pH significantly increased concentrations of labile P fractions in red soil, while there was no significant effect in fluvo-aquic soil (Fig. 5). This implies that increasing the pH in red soils could have promoted the distribution of P to the labile P fractions. In this study, the increment of Resin-P concentration in red soil amended with SSP was significantly less than that in fluvo-aquic soil, whereas there was no significant difference between the two soils amended with CM (Fig. 2). This may be attributed to the increase in soil pH and the decrease of P adsorption in red soil with CM, and the consequent improvement in soil P availability.

### P-NMR analysis of fluvo-aquic and red soil with different P sources

In this study, a large proportion of orthophosphate was found in soils with SSP, which is consistent with other previous reports^34,56^. In addition, orthophosphate in soil with SSP was significantly higher than that in soil with alternative P sources (Fig. 3), which is expected, at least in a short time, as SSP is readily soluble. PM and CM additions significantly increased concentrations of monoester P and inositol hexakisphosphate in both soils. Previous studies also reported that the application of CM and PM increased the content of soil phosphate monoester significantly, with the effect being more evident for PM than CM^57,58^. The proportion of inositol phosphate to total P is approximately 8% in CM^17,59^, but reaches up to 80% in PM^57,60^. Inositol phosphate may complex divalent and trivalent metal elements such as calcium, magnesium, zinc and iron to form extremely insoluble compounds, which can reduce the availability of P^61^. This may be an important factor explaining the higher availability of P in soils amended with CM than with PM. However, the mineralization of different Po fractions in soil and the associated mechanisms are still unclear. In this regard, studying the microbial processes of P transformation and its regulation in fluvo-aquic and red soils with alternative P sources would provide considerable insights into developing effective P management options for these soil types.

### Agricultural P management response to different alternative P sources

Achieving a sustainable P cycle requires both a reduction of chemical P input and an increase in alternative sources of P supply. Recycling and recovery of P from agricultural waste are essential to the sustainability of global P management^62–64^. The current study calls for closing the P cycle at the field scale by recycling manure, especially cattle manure, to maximize P use efficiency and minimize P losses in crop production systems. Although straw residual and bone meal are regarded as potential P storage, the P availability in these sources over the short term is nearly negligible. The availability of alternative P sources is strongly affected by soil type, and increasing pH of red soil is beneficial for the transformation of organic P into labile P fractions. In addition, the transformation of alternative P sources in soil could be affected by temperature, precipitation, tillage and cultivation system. This short-term incubation experiment was carried out under constant temperature and humidity conditions. Thus, the long-term effects of different alternative P sources on soil P transformation under field conditions should be further investigated. In summary, this study suggests that animal manure, especially CM, seems to be a good alternative P source with high P availability and is expected to play an important role in alleviating the limitation of P resources in agriculture.

## Conclusion

The interaction of soil types with different P sources determines the turnover and distribution of P among different soil P fractions. Compared to other alternative P sources, CM addition significantly increased the concentration of Olsen-P and distribution of soil labile P fractions. Therefore, CM is a superior alternative source for improving soil P availability in fluvo-aquic and red soils. P inputs from SSP, PM and CM were more strongly immobilized in red soil than fluvo-aquic soil, due to a reduction of labile P fractions in red soil. ^31^P-NMR study showed that amount of orthophosphate was the main factor affecting the availability of P from different P sources. The SEM analysis showed that the soil Olsen-P concentration was mainly affected by the labile P fraction. In addition, increasing the pH in red soils could have promoted the distribution of P to the labile P fractions. In summary, manure, especially cattle manure, can be an alternative effective source for P supply to alleviate chemical P limitation. Better understanding of P use efficiency of different P sources and their impact on yield and environmental impact under crop production conditions should be the next logical step.

## Materials and methods

### Experimental material and soil characteristics

The experiment was carried out in a growth chamber located in the Department of Plant Nutrition, College of Resources and Environment, Southwest University, Chongqing, China. A typical fluvo-aquic soil (alluvial soil in the US taxonomy) was collected in Quzhou, Hebei Province and a red soil (ultisols in the US taxonomy) was collected from a farmland in Shilin, Yunnan Province. The texture of fluvo-aquic soil is silt loam with 7.9% clay (< 2 μm), 55.3% silt (2–20 μm), and 36.8% sand (20–2000 μm). Other characteristics of fluvo-aquic soil were: 8.8 mg P kg^−1^ Olsen-P, 914.7 mg P kg^−1^ total P, pH 7.9 (water: soil ratio 2.5: 1), 7.7 g C kg^−1^ total organic carbon (TOC), 1.9 mg N kg^−1^ NH_4_^+^-N, 24.3 mg N kg^−1^ NO_3_^–^N, and 26.1 mg K kg^−1^ exchangeable potassium. Soil texture of red soil is clay with 47.5% clay (< 2 μm), 25.3% silt (2–20 μm), and 27.2% sand (20–2,000 μm). Other characteristics of red soil were: 38.2 mg P kg^−1^ Olsen-P, 1083.7 mg P kg^−1^ total P, pH 5.7 (water: soil ratio 2.5: 1), 20.2 g C kg^−1^ TOC, 2.4 mg N kg^−1^ NH_4_^+^-N, 42.7 mg N kg^−1^ NO_3_^–^N, and 78.2 mg K kg^−1^ exchangeable potassium. Before the experiment, both soils were air-dried and sieved (2 mm), then pre-incubated in the dark at 25 °C for 7 d, at a moisture level of 30.0% water holding capacity (WHC).

Five P sources including superphosphate (SSP), poultry manure (PM), cattle manure (CM), maize straw (MS) and cattle bone meal (CB) were used in the experiment. The total N-P-K contents were 16–20-32 g kg^−1^ of PM, 7–5-12 g kg^−1^ of CM, 7–5-8 g kg^−1^of MS and 37–93-1 g kg^−1^ of CB, respectively. The properties of PM were: 2.2 g kg^−1^ Olsen-P, pH 8.7 (water: soil ratio 2.5: 1), 346.9 g kg^−1^ TOC. The properties of CM were: 1.0 g kg^−1^ Olsen-P, pH 9.5, 242.9 g kg^−1^ TOC. The properties of MS were: 12.3 mg kg^−1^ Olsen-P, pH 8.4, 491.8 g kg^−1^ TOC. The properties of CB were: 2.0 g kg^−1^ Olsen-P, pH 8.0, 52.3 g kg^−1^ TOC.

### Experiment design

Treatments of this incubation experiment were a factorial design of two soil types (fluvo-aquic and red soils), and five different alternative P sources plus an unamended control (CK). The five P sources were SSP, PM, CM, MS and CB. The total application of P was 120 mg kg^−1^ for each treatment. The amounts of different amendments were determined according to the P content of different P sources. The total N and K_2_O inputs were supplemented with Ca (NO_3_)_2_ and KCl to 200 and 325 mg kg^−1^ in the soil of all treatments. The fertilizer amounts for each treatment are shown in Table S1. The air-dried soil and different alternative P sources were sieved with a 2 mm stainless steel sieve, and the soil was mixed with alternative P sources. Each treatment was replicated 36 times, and each experimental unit (a replicate) constituted a 200-ml cylinder plastic container that contained 100 g of soil. All containers were kept at 25℃ in an incubator for 70 days. During the whole incubation period, the gravimetric soil water content was kept at 30% WHC by weighing. A total of 432 experimental units (2 soil types × 6 P treatments × 36 repetitions) were used in this experiment. A large number of replicates allowed destructive sampling on each sampling date. Soil samples were taken for Olsen-P analysis at 0, 3, 7, 14, 21, 28, 35, 42 and 70 days after adding the alternative P sources. The soil P fractions were determined at 70 days after incubation (DAI).

### Sample analysis

#### Soil characterization and sequential P fractionation

Olsen-P was determined by the phosphomolybdate method after extraction using 0.5 mol L^−1^ NaHCO_3_, pH 8.5 (180 RPM, 25 °C) at 1:20 soil (W/V)^65^. After acid digestion with ammonium paramolybdate-vanadate reagent, soil total P was determined colorimetrically^66^. Soil TOC content was determined following a wet oxidation method with an acid mixture of K_2_Cr_2_O_7_ and H_2_SO_4_^67^. Soil samples were air-dried and ground to pass a 150 μm sieve and alternative P sources were frozen at -80 °C, lyophilized, and ground to pass a 150 μm sieve for the sequential extraction. The sequential extraction procedure proposed by Tiessen and Moir (1993) was used to determine different soil P fractions: Resin-P, NaHCO_3_-P, NaOH-P, dil.HCl-P, conc.HCl-P and Residual P. Pt in different extracts (NaHCO_3_-P, NaOH-P, conc.HCl-P) were determined using the ammonium persulfate digestion method^13^. P concentration of extracts was quantified colorimetrically^68^. The concentration of Po was calculated as the difference between total P (Pt) and inorganic P (Pi). Fig. S1 shows the detailed analysis process.

#### Pretreatment of samples for NMR analysis

Po analysis was also performed by NaOH-EDTA extraction followed by ^31^P-NMR analysis^69,70^. For the ^31^P-NMR analysis, soil samples on DAI 70 were ground and sieved through a 100-μm mesh. The soils were then extracted with a solution of 0.25 mol L^−1^ NaOH and 0.05 mol L^−1^ EDTA for 16 h at room temperature at a sediment: extract ratio of 1:10^58,71^. The solution pH was adjusted to 9.0 ± 1.0 by 1 M HCl, kept steady for 30 min, and then centrifuged at 12,000 g (20 ℃) for 30 min. The NaOH-EDTA solution was frozen and lyophilized for ^31^P-NMR analysis. This extract was re-dissolved in 2 mL of 1 mol L^−1^ NaOH solution for 2 h by vortex shaking, and the suspension was centrifuged at 12,000*g* (20 °C) for 30 min^72^. An aliquot (940 μL) of the supernatant was transferred into a 5-mm NMR tube, and added with a deuterated aqueous solution of methylenebisphosphonic acid-P, P′-disodium salt (MDP, Epsilon Chimie, Brest) as internal standard (δ = 16.62 ppm), to reach a final 2.65 mM concentration. For each treatment, three replicates were measured for NMR analyses^71^.

#### Solution ^31^P-NMR analysis

Solution ^31^P-NMR spectra were determined using a Bruker 600-MHz spectrometer (Bruker, AVANCE III, Switzerland) operated at 242.93 MHz at 25 °C. A power-gated decoupling pulse, a relaxation delay of 2 s, an acquisition time of 0.67 s and 4000 scans were set for the measurement. Chemical shifts were recorded relative to an 85% H_3_PO_4_ standard (δ = 0 ppm). All ^31^P spectra were baseline corrected and processed by MestReC software (v. 4.9.9.9). Signal areas were calculated by integrating the individual peaks resulting from a deconvolution process. With the peak of orthophosphate standardized at 6.18 ppm, signals were assigned to individual P compounds or compound classes based on publications^73–76^. The 0.2 mM methylene phosphonic acid (MDP) internal standard was used to dissolve samples for NMR analyses and calibrate the frequency axis, standardize data and perform a quantitative assessment of P compounds. The solution ^31^P NMR spectra of NaOH-EDTA extracts reflected the different alternative P sources in Fig. S3.

### Calculation method

The change in soil Olsen-P (ΔOlsen-P) and P fractions (ΔP fraction) was calculated as follows:

$$ \Delta {\text{Olsen - P}} = {\text{Olsen - P}}_{{{7}0}} {-}{\text{ Olsen - P}}_{0} $$

where Olsen-P_70_ is soil Olsen-P concentration on DAI 70 (mg kg^−1^), and Olsen-P_0_ is the soil Olsen-P concentration of initial soil.

$$ \Delta {\text{P}}\,{\text{fraction}}_{{\text{i}}} \, = \,{\text{P}}\,{\text{fraction}}_{{{\text{i7}}0}} \,{-}\,{\text{P}}\,{\text{fraction}}_{{{\text{i}}0}} $$

where P fraction_i70_ is the i-th P fraction concentration on DAI 70 (mg kg^−1^), and P fraction_i0_ is the i-th P fraction concentration of initial soil.

### Statistical analysis

NMR data were processed using the MestReNova package (V8.1.4 Mestrelab Research, Spain). Data were tested for the assumptions of normality and homoscedasticity using the Shapiro–Wilk’s test (*P* > 0.05). A two-way analysis of variance model was used to test the main and interactive effects of P source (df = 5) and soil type (df = 1) on the changes in soil Olsen-P and P fractions. Where treatment effects were significant, means were compared using the least significant difference (LSD) test at *P* < 0.05. All analyses were conducted using the SPSS software (SPSS 13, Chicago, IL, USA). The structural equation model (SEM) was used to identify the potential driving factors of the transformation of different P fractions following fertilizer applications in the two experimental soils using the IBM SPSS AMOS 22.0 (IBM Corporation 2013). Root-mean square-error of approximation (RMSEA) (< 0.08), chi-square (χ^2^) (χ^2^/df < 2), and the *P-value* of χ^2^ (*P* > 0.05) were used to evaluate the model fitting.

## Supplementary Information

Supplementary Information.

## Data Availability

The datasets generated during and/or analysed during the current study are available from the corresponding author on reasonable request.
