# Supplementary material for: Soil phosphorus availability and fractionation in response to different phosphorus sources in alkaline and acid soils: a short-term incubation study
Source: Sci Rep. 2023 Apr 7;13:5677. doi: 10.1038/s41598-023-31908-x (PMC10082179; doi:10.1038/s41598-023-31908-x)
Supplement: Supplementary file 1 — Supplementary Information. [file 41598_2023_31908_MOESM1_ESM.docx]

**Soil phosphorus availability and fractionation in response to different phosphorus sources to** **alkaline and acid soils: A short-term incubation study**

Yuan Wang^1,2^, Wei Zhang^1,2*^, Torsten Müller^3^, Prakash Lakshmanan^2,4,5^, Yu Liu^6^, Tao Liang^2,7^, Lin Wang^7^, Huaiyu Yang^1,2*^, Xinping Chen^1,2^

^1^ College of Resources and Environment, Academy of Agricultural Sciences, Key Laboratory of Efficient Utilization of Soil and Fertilizer Resources, Southwest University, Chongqing, 400716, China

^2^ Interdisciplinary Research Center for Agriculture Green Development in Yangtze River Basin, Southwest University, Chongqing, China

^3^ Institution of Crop Science, University of Hohenheim, Stuttgart, 70593, Germany

^4^ Sugarcane Research Institute, Guangxi Academy of Agricultural Sciences, Nanning 530007, China

^5^ Queensland Alliance for Agriculture and Food Innovation, University of Queensland, St Lucia 4067, QLD, Australia

^6^ College of life sciences, Zhejiang University, Zhejiang, 310058, China

^7^ Chongqing Academy of Agriculture Sciences, Chongqing 40000, China

*** Correspondence:**Huaiyu Yang
[yanghuaiyu@swu.edu.cn](mailto:yanghuaiyu@swu.edu.cn)

**Supplementary Material**

Supplementary Figures and Tables

##
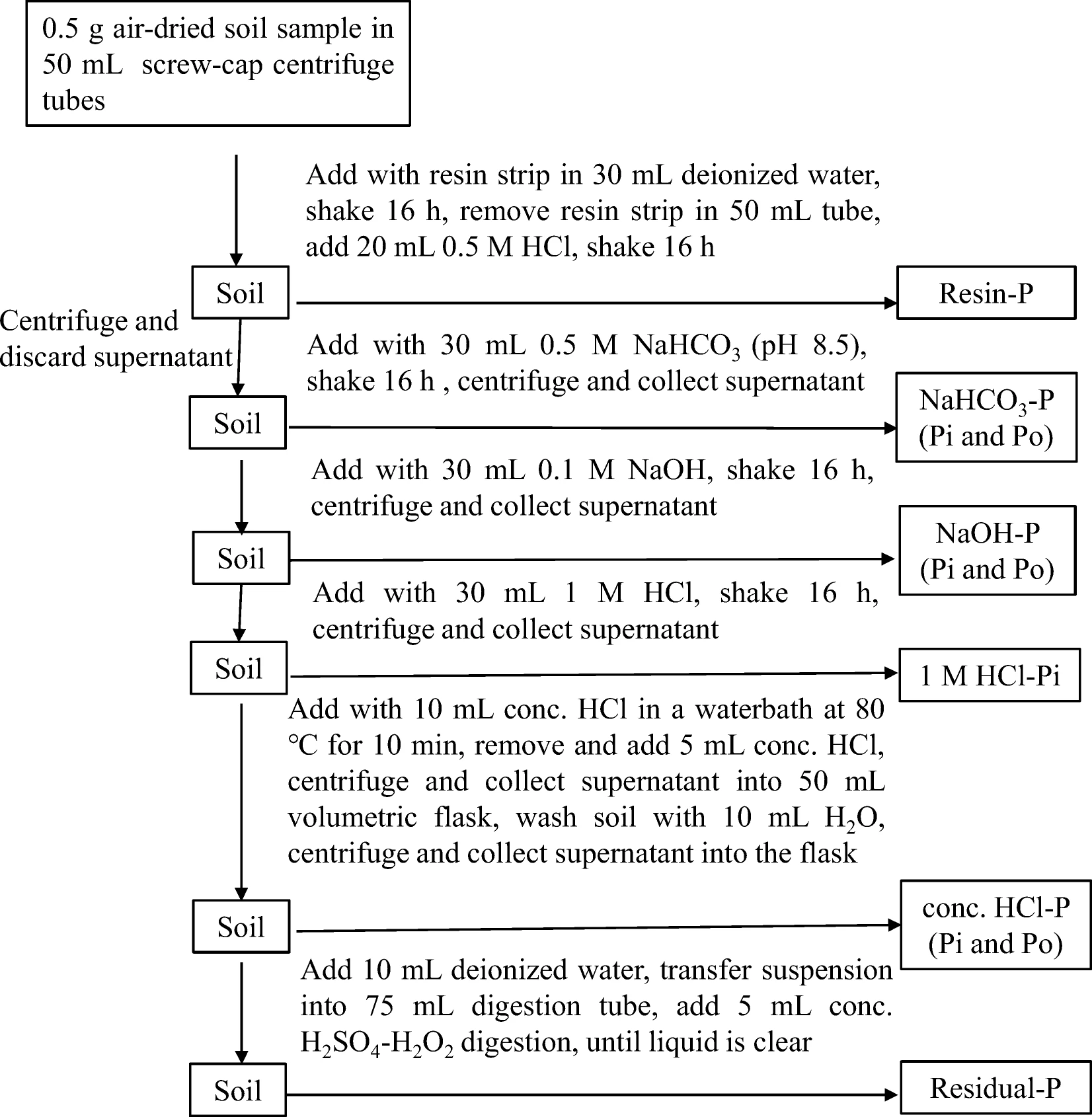


Fig. S1 Sequential soil phosphorus (P) fractionation method by Tiessen and Moir. Pi and Po, represent inorganic and organic P, respectively; conc. HCl indicates concentrated HCl.

**Table S1 The amounts of fertilizer added in each treatment**

| Treatments | Alternative P sources  (g·kg^-1^) | Ca(H_2_PO_4_)_2_  (g·kg^-1^) | Ca(NO_3_)_2_·4H_2_O  (g·kg^-1^) | KCl  (g·kg^-1^) |
| --- | --- | --- | --- | --- |
| CK | 0.00 | 0.00 | 1.69 | 0.62 |
| SSP | 0.00 | 0.45 | 1.69 | 0.62 |
| PM | 5.93 | 0.00 | 0.89 | 0.25 |
| CM | 26.55 | 0.00 | 0.18 | 0.01 |
| MS | 22.06 | 0.00 | 0.40 | 0.30 |
| CB | 0.65 | 0.00 | 1.29 | 0.62 |

Note: CK: Control; SSP: Ca(H_2_PO_4_)_2_; PM: Poultry Manure; CM: Cattle Manure; MS: Maize Straw; CB: Cattle Bone Meal.


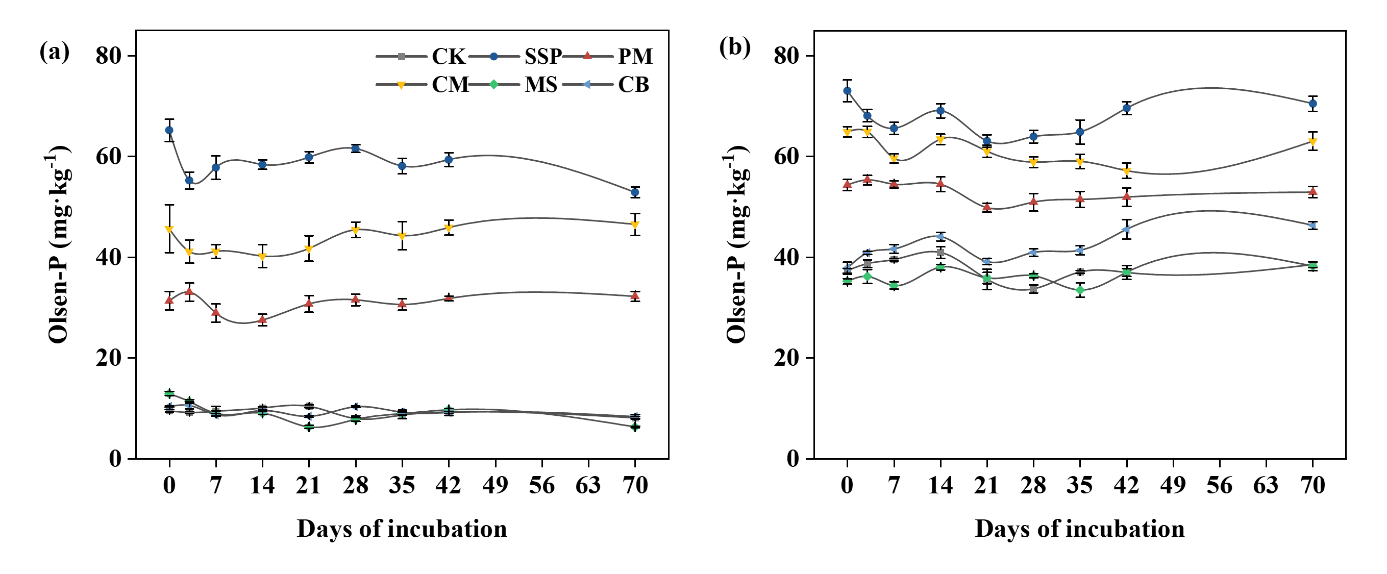


Fig. S2 Changes in Olsen-P concentrations in fluvo-aquic (a) and red soils (b) supplemented with different phosphorus sources during 70-day incubation. Values are means ± SE (n= 4). CK: Control; SSP: Ca(H_2_PO_4_)_2_; PM: Poultry Manure; CM: Cattle Manure; MS: Maize Straw; CB: Cattle Bone Meal. The same below.

Table S2 Phosphorus fractions in fluvo-aquic soil (FS), red soil (RS) and different alternative P sources as determined by the sequential soil P fraction method

| Samples | | Labile P  (mg kg^-1^) | | | | | |  | | Moderately labile P  (mg kg^-1^) | | | | | |  | | Sparingly labile P  (mg kg^-1^) | | | |  | | Non-labile P  (mg kg^-1^) | |
| --- | --- | --- | --- | --- | --- | --- | --- | --- | --- | --- | --- | --- | --- | --- | --- | --- | --- | --- | --- | --- | --- | --- | --- | --- | --- |
|  |  | Resin-P | | NaHCO_3_-Pi | | NaHCO_3_-Po | |  | | NaOH-Pi | | NaOH-Po | | dil.HCl-Pi | |  | | conc.HCl-Pi | | conc.HCl-Po | |  | | Residual-P | |
| Soils | FS | | 20.4 | | 11.7 | | 33.4 | |  | | 24.0 | | 34.4 | | 541.3 | |  | | 130.2 | | 35.2 | |  | | 71.0 |
|  | RS | | 80.6 | | 32.8 | | 46.2 | |  | | 297.8 | | 63.9 | | 36.8 | |  | | 390.1 | | 35.1 | |  | | 78.6 |
| Alternative P sources | PM | | 4359.0 | | 2144.7 | | 3342.6 | |  | | 612.6 | | 779.4 | | 8405.2 | |  | | 495.6 | | 532.0 | |  | | 69.0 |
|  | CM | | 1265.2 | | 1014.7 | | 858.2 | |  | | 389.1 | | 309.4 | | 314.3 | |  | | 282.2 | | 214.4 | |  | | 58.8 |
|  | MS | | 296.1 | | 12.3 | | 75.6 | |  | | 530.9 | | 455.4 | | 78.0 | |  | | 2526.2 | | 1136.0 | |  | | 148.8 |
|  | CB | | 3021.3 | | 2024.7 | | 3175.2 | |  | | 378.3 | | 266.6 | | 63201.5 | |  | | 9562.2 | | 8518.0 | |  | | 92.5 |

Note: FS: Fluvo-aquic soils; RS: Red soils; PM: Poultry Manure; CM: Cattle Manure; MS: Maize Straw; CB: Cattle Bone Meal. Labile P (Resin-P +NaHCO_3_-Pi +NaHCO_3_-Po), Moderately labile P (NaOH-Pi +NaOH-Po +1 M HCl-P), Sparingly labile P (conc. HCl-Pi + conc. HCl-Po) and Non-labile P (Residual-P) according to Crews and Brookes (2014) and Ahmed et al. (2019), the same below. dil. and conc. HCl indicates diluted and concentrated HCl, respectively.

Table S3 Phosphorus fractions in fluvo-aquic soil (FS) and red soil (RS) amended with different phosphorus sources as determined by the sequential soil P fraction method (Tiessen and Moir 1993) on DAI 70

|  | Treatments | Labile P  (mg kg^-1^) | | | Moderately labile P  (mg kg^-1^) | | | Sparingly labile P  (mg kg^-1^) | | Non-labile P  (mg kg^-1^) |
| --- | --- | --- | --- | --- | --- | --- | --- | --- | --- | --- |
|  |  | Resin-P | NaHCO_3_-Pi | NaHCO_3_-Po | NaOH-Pi | NaOH-Po | dil.HCl-Pi | conc.HCl-Pi | conc.HCl-Po | Residual-P |
| FS | CK | 18.8±1.3de | 11.4±1.2cd | 36.0±1.0c | 28.7±0.7c | 34.3±0.7c | 546.7±3.0c | 134.7±5.2c | 33.7±3.8c | 70.3±4.3b |
|  | SSP | 97.4±2.6a | 24.8±0.7b | 40.7±1.3bc | 40.0±2.9ab | 35.0±1.2c | 548.7±5.6bc | 137.0±4.9cd | 30.3±3cd | 71.0±1.2b |
|  | PM | 60.1±2.2c | 25.8±1.1b | 42.7±1.2ab | 35.7±1.9b | 41.0±1.5b | 566.7±8.1ab | 144.0±4.0de | 21.7±3.2de | 81.7±2.4a |
|  | CM | 70.7±5.3b | 45.3±1.5a | 43.7±1.5ab | 41.7±1.7a | 44.3±2.6b | 565.3±5.9ab | 132.3±3.7e | 18.3±0.9e | 67.0±1.5b |
|  | MS | 14.8±1.3e | 9.8±0.8d | 43.0±2.6ab | 27.0±1.0c | 54.0±1.7a | 536.7±6.8c | 163.0±3.6b | 46.7±3.2b | 66.3±2.7b |
|  | CB | 25.2±0.4d | 13.4±0.8c | 46.0±1.0a | 28.3±1.8c | 32.3±2.4c | 570.3±2.7a | 172.0±3.8a | 71.3±3.2a | 68.3±1.5b |
| RS | CK | 83.0±2.1d | 33.3±2.8b | 57.0±0.0c | 310.3±1.5c | 52.7±3.4bc | 43.7±0.9b | 372.7±3.5cd | 52.7±4.1c | 78.3±3.5ab |
|  | SSP | 139.7±4.6a | 53.7±4.1a | 67.7±2.0b | 332.7±3.3b | 56.0±3.2c | 48.7±1.3b | 376.3±5.2bc | 53.0±2.6c | 78.3±0.3ab |
|  | PM | 116.0±2.6b | 52.0±2.5a | 71.3±0.7b | 346.0±3.6a | 50.3±2.3bc | 53.0±1.2b | 361.3±3.7d | 66.7±3.2b | 84.7±2.3a |
|  | CM | 137.0±2.6a | 53.0±3.2a | 83.7±5.0a | 304.7±3.9c | 44.7±3.8c | 47.7±1.8b | 389.3±5.0b | 68.7±3.3b | 69.3±2.0c |
|  | MS | 93.3±2.3c | 30.0±1.5b | 56.0±3.2c | 310.0±1.5c | 104.3±1.5a | 46.3±1.5b | 385.7±3.5bc | 75.3±4.3b | 70.7±2.2c |
|  | CB | 93.3±3.8c | 38.3±0.7b | 72.0±3.5b | 283.7±1.2d | 34.7±1.8d | 76.7±6.4a | 411.0±3.5a | 127.3±3.2a | 72.3±1.7bc |

Note: FS: Fluvo-aquic soils; RS: Red soils; CK: Control; SSP: Ca(H_2_PO_4_)_2_; PM: Poultry Manure; CM: Cattle Manure; MS: Maize Straw; CB: Cattle Bone Meal. Labile P (Resin-P +NaHCO_3_-Pi +NaHCO_3_-Po), Moderately labile P (NaOH-Pi +NaOH-Po +1 M HCl-P), Sparingly labile P (conc. HCl-Pi + conc. HCl-Po) and Non-labile P (Residual-P) according to Crews and Brookes (2014) and Ahmed et al. (2019). Values are means of four replicates ± standard errors. dil. and conc. HCl indicates diluted and concentrated HCl, respectively. In each column within a same soil type, means followed by a same lowercase letter are not significantly different at the 5% probability level according to the LSD test.


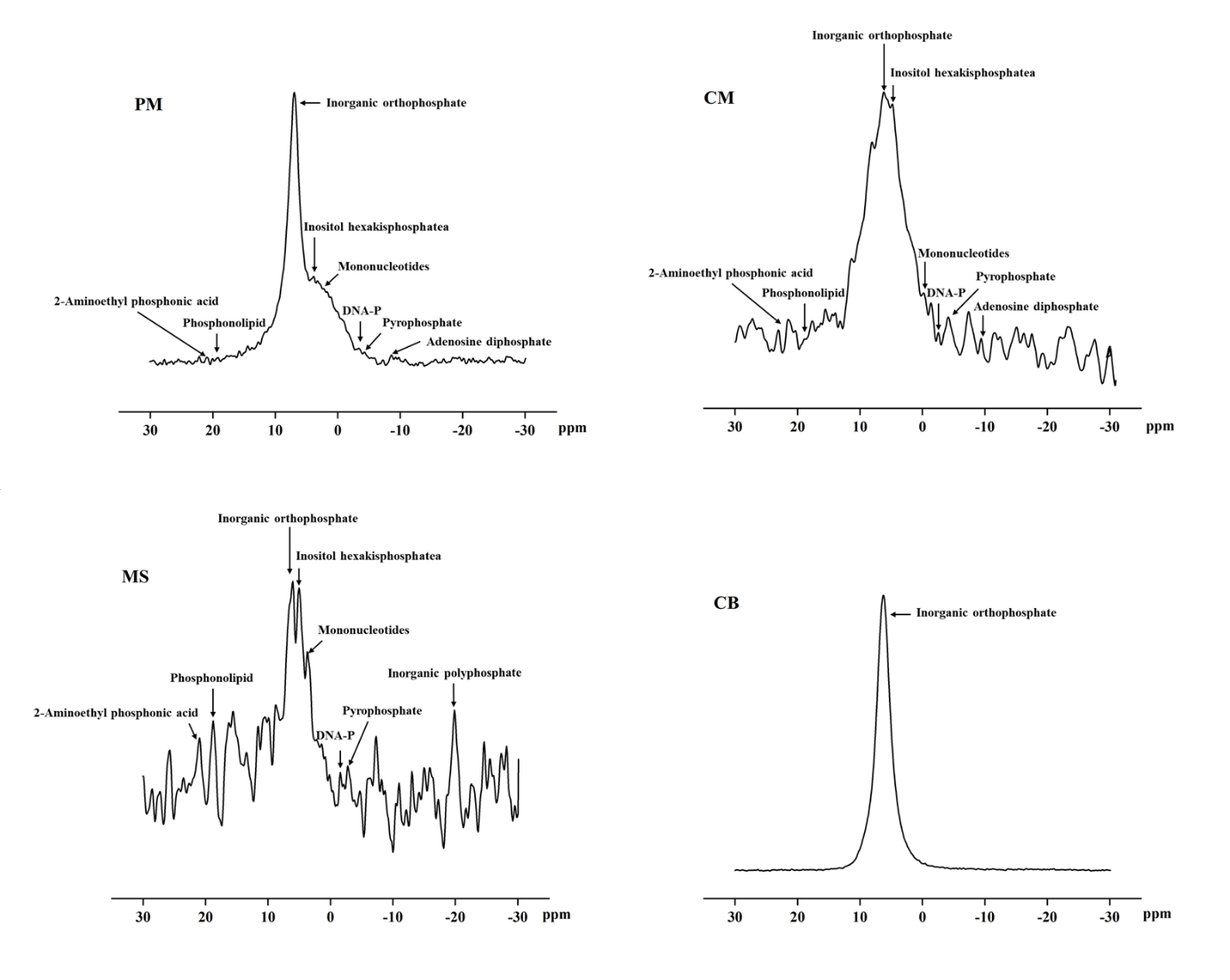


Fig. S3 Liquid ^31^P NMR spectra of NaOH-EDTA extracts of different P compounds. In the upper spectrum, the shift positions of the different P compounds are indicated. PM: Poultry Manure; CM: Cattle Manure; MS: Maize Straw; CB: Cattle Bone Meal


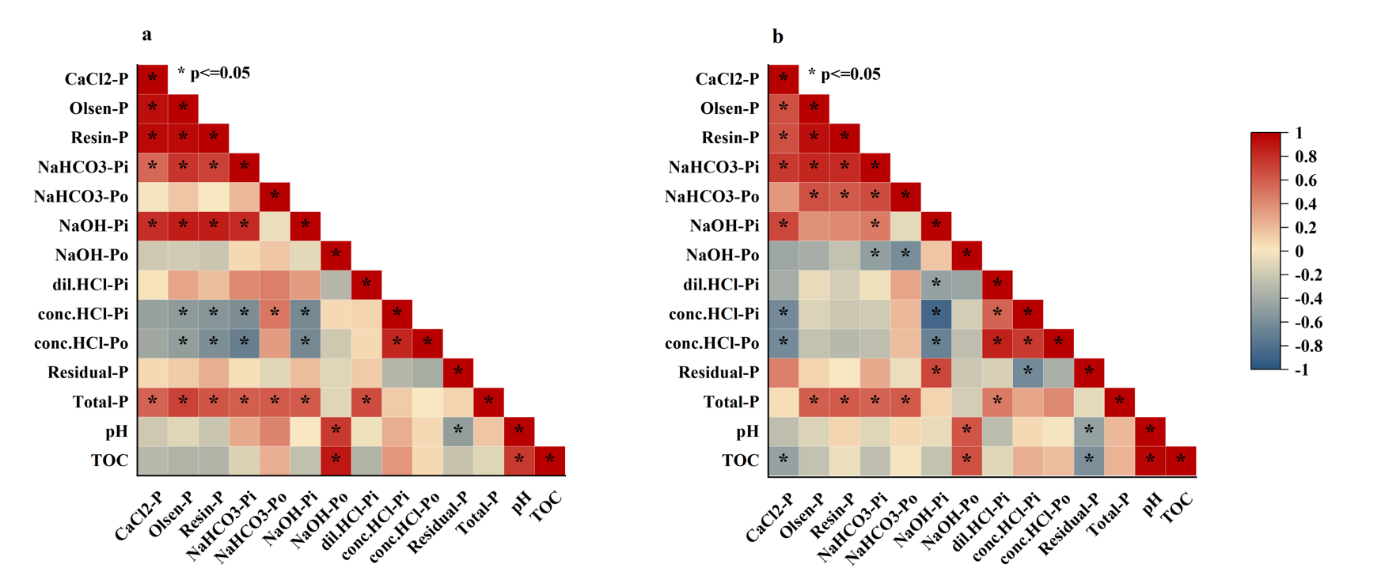


Fig. S4 Relationships between Olsen-P concentration and each soil P fractions and other soil properties in fluvo-aquic (a) and red soils (b) with different alternative P sources on DAI 70. * indicate significant regressions at *P* < 0.05.
